# Supplementary material for: Opposing actions of co-released GABA and neurotensin on the activity of preoptic neurons and on body temperature
Source: bioRxiv. 2024 Apr 17:2024.04.15.589556. Preprint. [Version 1] doi: 10.1101/2024.04.15.589556 (PMC11042348; doi:10.1101/2024.04.15.589556)
Supplement: Supplement 1 [file NIHPP2024.04.15.589556v1-supplement-1.pdf]

bioRxiv preprint doi: https://doi.org/10.1101/2024.04.15.589556; this version posted April 17, 2024. The copyright holder for this preprint (which was not certified by peer review) is the author/funder, who has granted bioRxiv a license to display the preprint in perpetuity. It is made available under aCC-BY 4.0 International license.

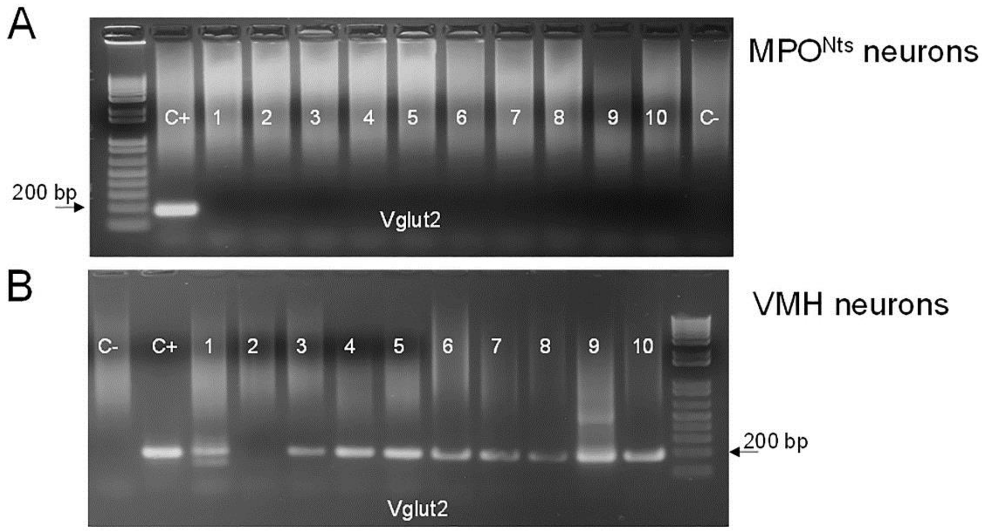

Figure1 Suppl. Lack of Vglut2 expression in MPO^{Nts} neurons

A. Single cell RT/PCR analysis of Vglut2 expression. Representative results from 10 MPO$^{Nts;hChR2}$ neurons.

B. Vglut2 expression in single ventromedial hypothalamus (VMH) neurons. Vglut2 transcripts were detected in 9 out of 10 neurons.

A,B. The expected size of the PCR product is 184 base pairs. Negative (−) control was amplified from a harvested cell without reverse-transcription, and positive control (+) was amplified using 1 ng of hypothalamic mRNA.

bioRxiv preprint doi: https://doi.org/10.1101/2024.04.15.589556; this version posted April 17, 2024. The copyright holder for this preprint (which was not certified by peer review) is the author/funder, who has granted bioRxiv a license to display the preprint in perpetuity. It is made available under aCC-BY 4.0 International license.

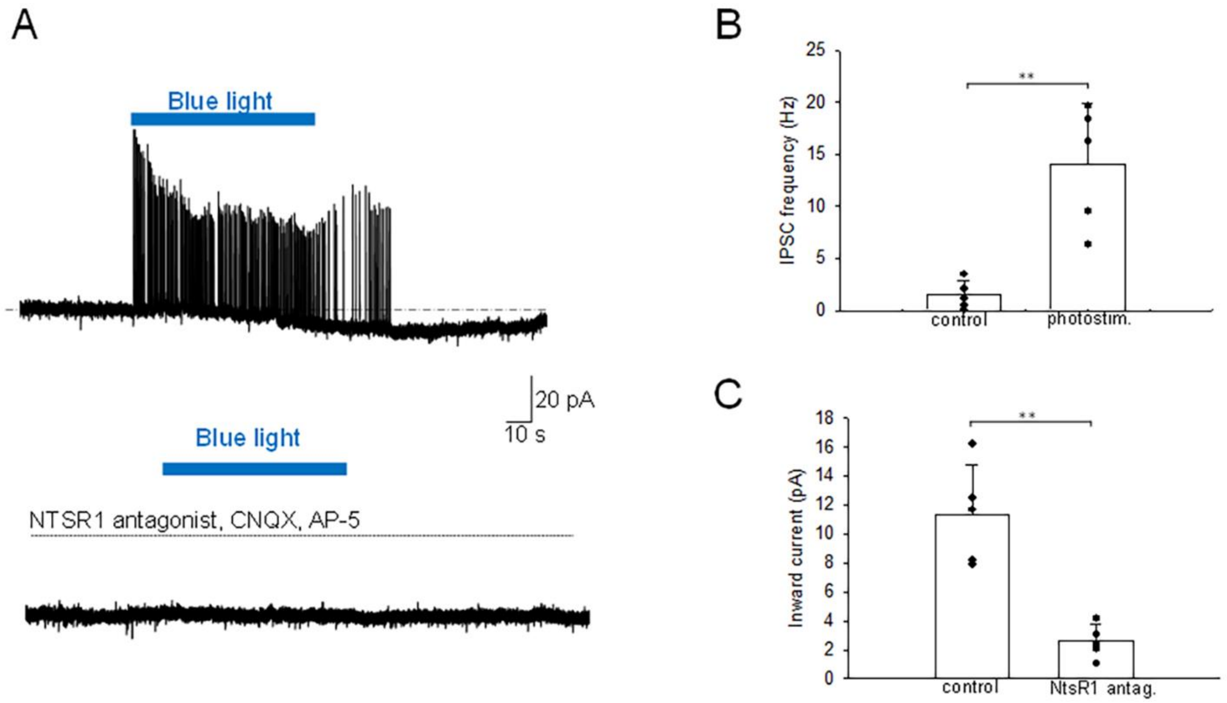

Figure 3 Suppl. Optogenetic stimulation of MPO$^{Nts;hChR2}$ neurons from females increases the frequency of IPSCs and activates an inward current in nearby MPO neurons

A. Optogenetic stimulation of a MPO$^{Nts;hChR2}$ neuron activates IPSCs and an inward current in a nearby MPO neuron (upper trace). In the presence of CNQX (20 µM), AP-5 (50 µM), Gabazine (5 µM) and the NtsR1 antagonist SR48692 (100 nM) light stimulation was without effect (lower trace). The neuron was held at -50 mV.

B,C. Bar charts summarizing the increase in the frequency of IPSCs (**B**) and the amplitude of the inward current (**C**) recorded in MPO neurons in response to optogenetic stimulation of several MPO$^{Nts;hChR2}$ neurons. **B.** The IPSCs frequency increased from1.5±1.4 Hz to 14.1±5.8 Hz in response to photostimulation (one-way ANOVA F(1,9)=22.4, $p=1.4x10^{-3}$). **C**. The average inward current activated by optogenetic stimulation decreased from 11.3±3.4 pA to 2.6±1.2 pA in the presence of the NtsR1 antagonist SR48692 (100 nM) (one-way ANOVA (F(1,9)=28.9, $p=6.6x10^{-4}$). Bars represent means ± S.D. Data pooled from n=5 neurons.

bioRxiv preprint doi: https://doi.org/10.1101/2024.04.15.589556; this version posted April 17, 2024. The copyright holder for this preprint (which was not certified by peer review) is the author/funder, who has granted bioRxiv a license to display the preprint in perpetuity. It is made available under aCC-BY 4.0 International license.

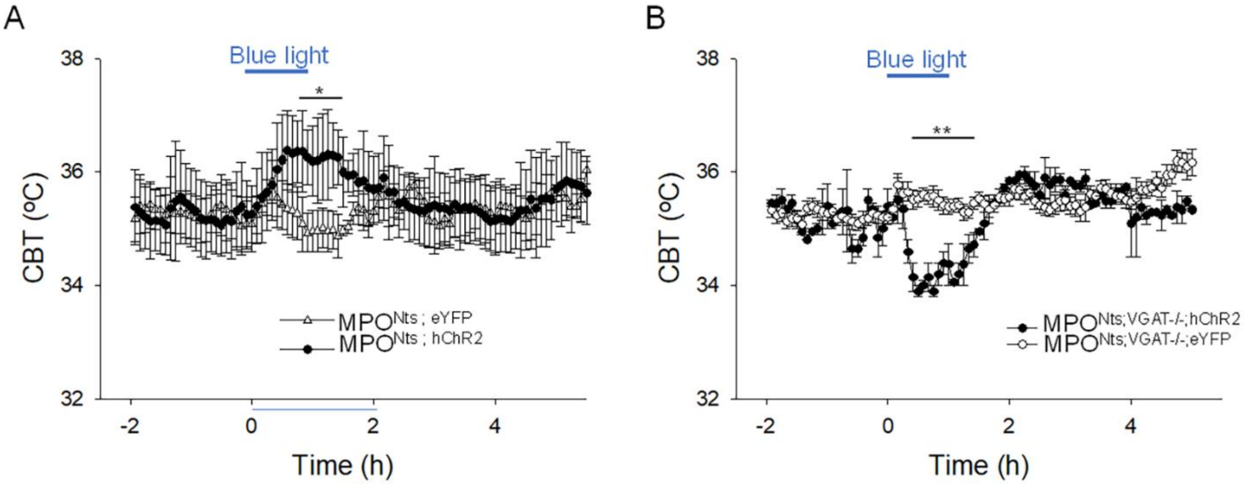

Figure 4 Suppl. In female mice optogenetic activation of MPO$^{Nts;hChR2}$ neurons induces hyperthermia while optogenetic activation of MPO$^{Nts;VGAT-/-;hChR2}$ neurons induces hypothermia

A. Optogenetic stimulation of MPO$^{Nts;hChR2}$ neurons (●) *in vivo* for 1 hour (blue light) induced a hyperthermia of 1.31±0.65 °C relative to control (∆). The response was statistically different to the response to photostimulation of control MPO$^{Nts;eYFP}$ mice (∆) (one-way repeated measures ANOVA, $F(1,110)=5.7$, $p=1.9\times10^{-2}$, followed by Man-Whitney U tests for each time point, * $P<0.05$).

bioRxiv preprint doi: https://doi.org/10.1101/2024.04.15.589556; this version posted April 17, 2024. The copyright holder for this preprint (which was not certified by peer review) is the author/funder, who has granted bioRxiv a license to display the preprint in perpetuity. It is made available under aCC-BY 4.0 International license.

B. Optogenetic stimulation of $MPO^{Nts;VGAT-/-;hChR2}$ neurons (●) *in vivo* for 1 hour (blue light) induced a hypothermia of 1.69±0.22 °C relative to control (∆). The response was statistically different to the response to photostimulation of control $MPO^{Nts;VGAT-/-;eYFP}$ mice (∆) (one-way repeated measures ANOVA, $F(1,83)=9.9$, $p=2.2 \times 10^{-3}$, followed by Man-Whitney U tests for each time point, ** $P<0.01$).

A,B. The points represent averages±S.D. through the 7h recording period. Experiments were carried out in parallel in groups of 6 female mice.

bioRxiv preprint doi: https://doi.org/10.1101/2024.04.15.589556; this version posted April 17, 2024. The copyright holder for this preprint (which was not certified by peer review) is the author/funder, who has granted bioRxiv a license to display the preprint in perpetuity. It is made available under aCC-BY 4.0 International license.

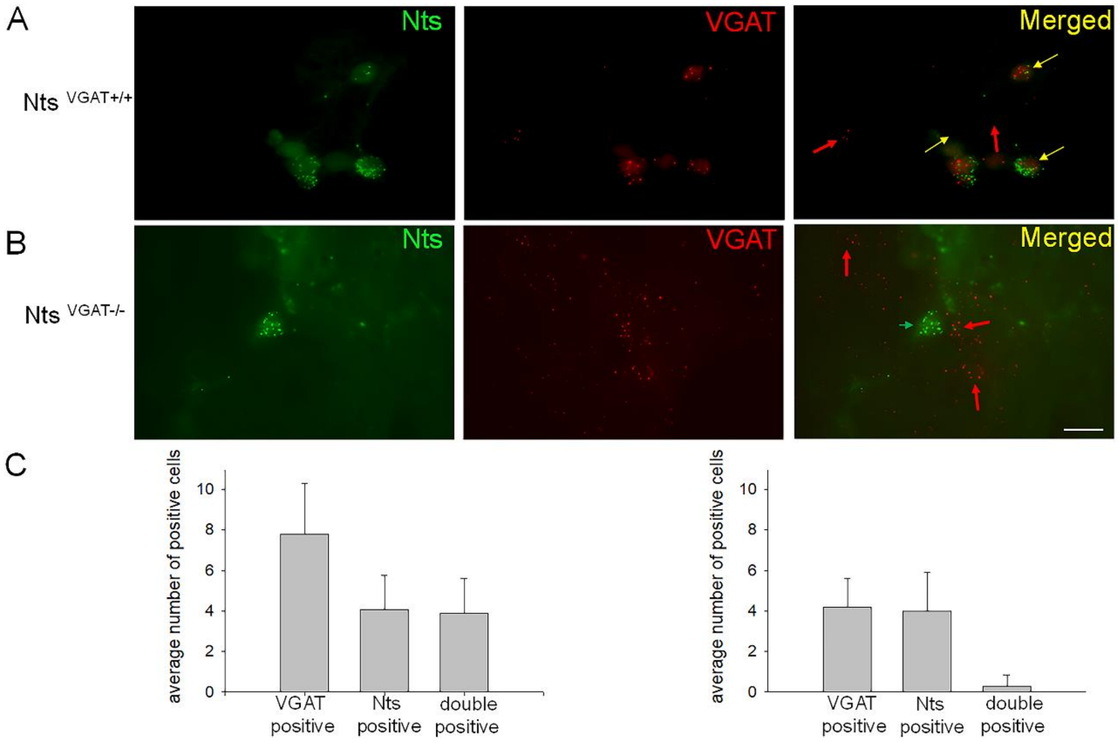

Figure 5 Suppl. Expression of VGAT transcripts in preoptic slices from Nts$^{VGAT-/-}$ and Nts$^{VGAT+/+}$ male mice.

A,B. Representative images of VGAT (red) and Nts (green) transcripts visualized using RNAscope technology in preoptic slices from Nts$^{VGAT+/+}$ (**A**) and Nts$^{VGAT-/-}$ mice (**B**).

A. Nts transcripts (green, left) are present in 3 out of 5 VGAT positive cells (red, middle) as indicated by their superimposed images (right, yellow arrows). Two other VGAT expressing cells do not express Nts transcripts (right, red arrows). **B.** Nts transcripts (green, left) are present in 2 cells while VGAT transcripts (red, middle) are present in 6 cells (middle). In Nts$^{VGAT-/-}$ tissue the VGAT positive cells (right, red arrows) do not co-express Nts transcripts. **A,B.** The scale bar represents 10 µm.

C. Bar charts summarizing the average number positive cells for the respective transcripts in a randomly selected field of view in Nts$^{VGAT+/+}$ (left) and Nts$^{VGAT-/-}$ (right) preoptic slices. Data were averaged from 15 randomly selected fields of view and 3 different mice for each genotype.

bioRxiv preprint doi: https://doi.org/10.1101/2024.04.15.589556; this version posted April 17, 2024. The copyright holder for this preprint (which was not certified by peer review) is the author/funder, who has granted bioRxiv a license to display the preprint in perpetuity. It is made available under aCC-BY 4.0 International license.

Supplementary Table 1. PCR primers for the genes studied

	Gene	External Primers	Ampli-con size	Internal Primers	Ampli-con size

bioRxiv preprint doi: https://doi.org/10.1101/2024.04.15.589556; this version posted April 17, 2024. The copyright holder for this preprint (which was not certified by peer review) is the author/funder, who has granted bioRxiv a license to display the preprint in perpetuity. It is made available under aCC-BY 4.0 International license.

1	Neurotensin (Nts)	F: “AGGCCCTACATTCTCAAGAG”<br>R: “CATTGTTCTGCTTTGGGTTA”	Bp:398	F: “GGGGTTCCTACTACTACTGA”<br>R: “CATCACATCCAATAAAGCAC”	Bp:149
2	Slc32a1 (VGAT)	F:“ GTCACGACAAACCCAAAGATCAC”<br>R: “**GTTGTTCCCTCATCATCTTCGCC**”	Bp:137		
3	PACAP	F: “ATGTCGCCCACGAAATCCTTAAC”<br>R: “GTCCGAGTGGCGTTTGGTA”	Bp:158		
4	Vglut2	F:“CTGGATGGTCGTCAGTATTTTATG”<br>R:“ATGAGAGTAGCCAACAACCAGAAG”	Bp:503	F:“GCAGGAGCTGGACTTTTTATTTAC”<br>R: “TAGTTGTTGAGAGAATTTGCTTGC”	Bp:186

Supplementary Table 2. P-values for Tukey’s test comparisons among groups (Fig 2B)

Pair	P-value
x_1-x_2	0.0001476
x_1-x_3	0.0003445
x_1-x_4	0.0005521
x_1-x_5	0.007513
x_1-x_6	0.01494
x_2-x_3	1.676e-8
x_2-x_4	1.751e-7
x_2-x_5	1.178e-8
x_2-x_6	1.447e-7
x_3-x_4	0.006143
x_3-x_5	0.0007085
x_3-x_6	0.002418
x_4-x_5	0.0003605
x_4-x_6	0.000312
x_5-x_6	0.2656

Supplementary Table 3. P values of the Tukey’s test comparisons among groups (Fig 5B)

bioRxiv preprint doi: https://doi.org/10.1101/2024.04.15.589556; this version posted April 17, 2024. The copyright holder for this preprint (which was not certified by peer review) is the author/funder, who has granted bioRxiv a license to display the preprint in perpetuity. It is made available under aCC-BY 4.0 International license.

Columns	P-value
x1-x2	7.1×10^{-5}
x1-x3	0.7237
x2-x3	7.82×10^{-6}

Supplementary Table 4. P values of the Tukey's test comparisons among groups (Fig 7F)

Pair	P-value
x1-x2	0.044
x1-x3	0.4212
x1-x4	0.0201
x2-x3	0.543
x2-x4	0.978
x3-x4	0.3303

Supplementary Table 5. P values of the Tukey's test comparisons among groups (Fig 7G)

Pair	P-value
x1-x2	0.0001877
x1-x3	0.05677
x1-x4	0.001055
x2-x3	0.04938
x2-x4	0.8145
x3-x4	0.2338

Supplementary Table 6. P values of the Tukey's test comparisons among groups (Fig 7H)

Pair	P-value
x1-x2	0.003388

bioRxiv preprint doi: https://doi.org/10.1101/2024.04.15.589556; this version posted April 17, 2024. The copyright holder for this preprint (which was not certified by peer review) is the author/funder, who has granted bioRxiv a license to display the preprint in perpetuity. It is made available under aCC-BY 4.0 International license.

x1-x3	0.01139
x1-x4	0.04768
x2-x3	0.9318
x2-x4	0.5657
x3-x4	0.8872

Supplementary Table 7. P values of the Tukey's test comparisons among groups (Fig 7I)

Pair	P-value
x1-x2	0.001157
x1-x3	0.00516
x1-x4	0.2825
x2-x3	0.8803
x2-x4	0.04888
x3-x4	0.1844
